# No Smoke without Tobacco: A Global Overview of Cannabis and Tobacco Routes of Administration and Their Association with Intention to Quit

**DOI:** 10.3389/fpsyt.2016.00104

**Published:** 2016-07-05

**Authors:** Chandni Hindocha, Tom P. Freeman, Jason A. Ferris, Michael T. Lynskey, Adam R. Winstock

**Affiliations:** ^1^Clinical Psychopharmacology Unit, University College London, London, UK; ^2^Institute for Social Science Research, University of Queensland, QLD, Australia; ^3^Department of Addiction, Institute of Psychiatry, Psychology and Neuroscience, King’s College London, London, UK; ^4^South London and the Maudsley NHS Trust, London, UK; ^5^Global Drugs Survey Ltd., London, UK

**Keywords:** cannabis, tobacco, marijuana, routes of administration, co-administration, inhalation

## Abstract

Cannabis and tobacco are common drugs of abuse worldwide and are often used in combination through various routes of administration (ROAs). Here, we aimed to provide an overview of how cannabis and tobacco routes varied across countries and assess the impact of tobacco-based ROAs on motivation to use less cannabis, and less tobacco, in different models. A cross-sectional online survey (Global Drugs Survey 2014) was completed by 33,687 respondents (mean age = 27.9; % female = 25.9) who smoked cannabis at least once in the last 12 months. Most common ROA, frequency of cannabis/tobacco use, and questions about motivation to use less cannabis/tobacco were recorded. Tobacco-based ROA were used by 65.6% of respondents. These were most common in Europe (77.2–90.9%) and Australasia (20.7–51.6%) and uncommon in the Americas (4.4–16.0%). Vaporizer use was most common in Canada (13.2%) and the United States (11.2%). Using a non-tobacco ROA was associated with a 10.7% increase in odds for “desire to use less” tobacco (OR: 1.107, 95% CI: 1.003, 1.221), 80.6% increase in odds for “like help to use less tobacco” (OR: 1.806, 95% CI: 1.556, 2.095), and a 103.9% increase in the odds for “planning to seek help to use less tobacco” (OR: 2.039, 95% CI: 1.638, 2.539), in comparison to using a tobacco-based ROA. Associations between ROA and intentions to use less cannabis were inconsistent. Results support considerable global variation in cannabis and tobacco ROA. Tobacco routes are common, especially “joints with tobacco,” especially in Europe, but not in the Americas. Non-tobacco-based routes are associated with increased motivation to change tobacco use. Interventions addressing tobacco and cannabis need to accommodate this finding and encourage non-tobacco routes.

## Introduction

Cannabis and tobacco are two of the world’s most commonly used drugs, with recent prevalence statistics suggesting one billion people worldwide smoke tobacco, equating to 22.6% of adults. Cannabis is also the most commonly used illicit drug worldwide with 3.5% of adults (174 million people) using cannabis, with highest rates of use in Oceania (10.3%) (1).

Much of the research investigating cannabis and tobacco use suffers from being unable to detangle the association of cannabis with tobacco (simultaneous use; for example, in joints or blunts) and using cannabis and tobacco (concurrent use; cannabis smoking and cigarette smoking separately), and there is a paucity of data available to detangle this. The distinction becomes important as those using cannabis with tobacco seem to have higher rates of DSM-IV cannabis abuse, even when adjusting for cannabis use and cigarette smoking (i.e., the independent use of both cannabis and tobacco) (2). Agrawal et al. (2) found that those who used smoked tobacco, in comparison to smokeless forms, were more likely to develop cannabis dependence. This may represent either a physiological adaption to “smoking” or may be related to cultural or social factors surrounding routes of administration (ROAs) (3).

Routes of administration, and especially inhalation ROAs, are important because the aerorespiratory alterations produced by smoking (e.g., cigarettes), may enable processes in favor of (e.g., cannabis) inhalation (3). ROAs are also important as they can alter the subjective experience of the drug (4). Use of tobacco (e.g., in joints) may confer a “practical advantage” to cannabis users, in as much as tobacco can increase the amount of delta-9-tetrahydrocannabinol (THC) inhaled per gram by up to 45% (5), and preclinical research suggests this tobacco pretreatment may increase the reinforcing properties of THC (6). Furthermore, smoking cannabis with inexpensive tobacco is economically advantageous as it dilutes the cost of the more expensive cannabis. Finally, it has been hypothesized that tobacco compensates for adverse cognitive and affective consequences of cannabis (7, 8). Therefore, ROAs may play a large role in the use of both drugs and the effects of cannabis may indeed vary by ROA. Given the scale of use of cannabis and tobacco, there has been little effort toward prevention and treatment; however, initial results seem promising (9, 10).

This study provides a worldwide summary of ROAs for recreational use of cannabis using data from the Global Drug Survey (GDS) 2014. Access to a worldwide sample allows us to collect data from participants who use various ROAs, which is not possible in single country samples (which are generally homogenous). Moreover, we aim to investigate if ROAs influence desire and motivation to quit cannabis and tobacco, after adjusting for the confounding effects of frequency of both drugs and demographic variables. Those who smoke cannabis and tobacco have poor cessation outcomes (11), and cannabis use itself may act as a barrier to change as it is related to a cannabis amotivational syndrome (at least acutely)^1^. Motivations related to cessation are important preparatory steps in the quitting process (12) and are the key to some therapy styles, such as motivational enhancement therapy (13). Moreover, therapies designed to support motivation to quit have an impact on both cigarette smoking cessation (14) and cannabis cessation (15). We hypothesized that non-tobacco ROAs (in comparison to tobacco ROAs) will be associated with increased motivation to quit (i) cannabis and (ii) tobacco.

## Materials and Methods

### Design and Participants

The GDS is an anonymous, self-nominating, cross-sectional online survey of drug use, conducted annually, in partnership with global media partners. Participants are recruited through onward promotion and online social networks on websites, such as The Guardian, MixMag, The Ziet, and other International publications. Demographic information is also collected, including age, gender, and country of residence. Data were collected throughout November 2013 and December 2013.

#### Sample

A total of 74,864 responses were received. The number of respondents varied across countries; therefore, data were only included from countries with ≥500 respondents (*n* = 70,977; 94.8% of the sample) because of reliability considerations. Furthermore, analysis was restricted to respondents who had used cannabis at least once, in the past 12 months (*n* = 33,687, 47.4% of the whole sample). This is a low threshold for cannabis use; however, we sought to capture a wide range of variation in cannabis use (see Table S1 in Supplementary Material for a replication of this analysis with regular cannabis users). This sample was selected specifically to be cannabis users, and within this sample, we were interested in varying levels of tobacco from no use at all, i.e., vaporizer use to heavy use, i.e., smoking cannabis and tobacco joints. Moreover, there was no analogous threshold for tobacco as not all cannabis users smoke tobacco, and we also wanted to capture this. All participants confirmed that they were 18+ years and gave informed consent. This study was approved by the joint South London and Maudsley NHS and Institute of Psychiatry Ethics Committee. This study was carried out in accordance with the Declaration of Helsinki.

### Statistical Analysis

All analyses were conducted in SPSS version 23 (IBM). Valid percentages are reported rather than absolute values for descriptive statistics to account for missing data. A list of assessments can be found in Table 1. Binary logistic regression was used to assess the effects of cannabis and tobacco, independently and combined, on six outcome variables that were considered a proxy to possible quitting behavior stages, as they align with the Stages of Change model (12) with each question requiring more commitment than the last (see cannabis assessments – items 2, 3, and 4 and tobacco assessments – items 1, 2, and 3). These were analyzed in different models, and “unsure” responses were removed from the analysis (there were a total 759 unsure response for “seek help to use less cannabis” and 1819 unsure responses to “seek help to use less tobacco”). Participants were not required to answer every question leading to missing data (see Missing Data); complete case analysis was used. As each of the motivation-based outcome questions were binary, analysis was undertaken using logistic regression. We included the following *a priori* variables to adjust for possible confounding variables: gender (binary; female as reference group) and age (in years). We then added frequency of cannabis use, frequency of tobacco use, and frequency of “cannabis mixed with tobacco” use. Finally, we used “most common ROAs,” which was coded dichotomously as either *tobacco ROAs* (reference group) (includes joint, blunt, pipe, bong/water pipe, and vaporizer with tobacco) or *non-tobacco ROAs* (includes joint, blunt, pipe, and bong/water pipe without tobacco). Adjusted odds ratios (aORs) and 95% confidence internals (95% CI) are reported for each model. An odds ratio (OR) >1 is suggestive of non-tobacco routes being associated with increased motivation to change in comparison to tobacco routes. Odds ratios <1 suggest non-tobacco routes being associated with reduced motivation to change in comparison to tobacco routes.

**Table 1:** **List of assessments**.

Drug history: for cannabis only, tobacco only, and tobacco mixed with cannabis	Ever used? (yes/no) Age of first use? (in years) Used in the last 12 months? (yes/no) Number of days used in the last 30 days?^a^ Used in the last 7 days? (yes/no)
Route of administration	Which is the most common way you currently use cannabis? (Select one): (a) Smoked in joint with tobacco (b) Smoked in blunt with tobacco (c) Smoked in pipe with tobacco (d) Smoked in bong/water pipe with tobacco (e) Smoked in joint without tobacco (f) Smoked in blunt without tobacco (g) Smoked in pipe without tobacco (h) Smoked in bong/water pipe without tobacco (i) Smoked using “bucket bong” (j) Smoked using hot knife (k) Using vaporizer (l) Eating it in food (m) Drinking in tea/infusion (n) Other
Impact of drug use	Typically, on a day that you use cannabis, how much cannabis do you use? (in grams) How would you rate the overall negative effects when high (rated between 1 and 10) How would you rate the overall pleasurable effect when high (rated between 1 and 10)
Intention to use less of each drug: for cannabis only and tobacco only	Would you like to use less cannabis/tobacco over the next 12 months? (yes/unsure/no) Would you like help to use less cannabis/tobacco over the next 12 months? (yes/unsure/no) Are you planning to seek help to use less cannabis/tobacco over the next 12 months? (yes/unsure/no)

*The structure of the GDS is personalized based on this drug use history; therefore, if the respondent has never used cannabis, for example, they would not have the opportunity to answer questions regarding cannabis*.

*^a^Used in Table 3 as DPM cannabis, tobacco, and tobacco with cannabis*.

#### Exploratory Analysis

We also investigated the association of ROAs with age and gender. We conducted exploratory analyses using the Brown–Forsyth *F*-test, which is robust to violations in homogeneity of variance (and that of unequal sample sizes) to investigate the association between ROA (non-tobacco ROA vs. tobacco ROA), frequency of cannabis use, frequency of tobacco use, quantity of cannabis use, the negative impact of cannabis use, the pleasurable effects of cannabis use, and age of first tobacco use. Moreover, we compared those who used a vaporizer as a non-tobacco ROA and those who use other non-tobacco ROAs on frequency of tobacco use.

#### Missing Data

Respondents were not required to answer every question. There were 191 missing responses for “Would you like to use less cannabis over the next 12 months?” 14,484 missing responses for “Would you like help to use less cannabis over the next 12 months?” and 14,456 missing responses for “Are you planning to seek help to use less cannabis over the next 12 months?” Missing data for “Would you like to use less tobacco over the next 12 months” was 3855 responses, “Would you like help to use less cannabis over the next 12 months” was 10,547 responses, and for “Are you planning to seek help to use less tobacco over the next 12 months” there were 10,432 missing responses. We did not impute the data, but instead, used valid percentages rather than absolute percentages where missing data occurred.

#### Sensitivity Analysis

We did not include the very infrequently chosen non-tobacco routes of “bucket bong,” “hot knife,” “in food,” “in drink,” or “other” (2.4% total). However, we did repeat the analysis with these variables combined with non-tobacco routes and replicated the results presented here. We also repeated the results by removing “cannabis mixed with tobacco,” as it was highly multicollinear with frequency of cannabis use; however, we report results with the frequency of “cannabis mixed with tobacco” predictor as it replicated the result without this variable. Finally, we replicated the results in a subpopulation of regular cannabis users who used cannabis >100 days in the last 12 months (see Table S1 in Supplementary Material).

## Results

### Global Overview of Cannabis and Tobacco Use

Inspection of Table 2 indicates that the final sample were young, with a mean (SD) age of 27.86 (10.39) years. Across individual countries, mean (SD) age ranged from 22.38 (5.95) in The Netherlands to 32.95 (11.52) in Australia. 25.86% of all respondents were female. Gender was skewed toward more male respondents from The Netherlands (41.6% female) to Denmark (19.1% female).

**Table 2:** **Cannabis and tobacco routes of administration by country**.

				Routes of administration with tobacco (%)						Routes of administration without tobacco (%)						
Country	Total *N*	*N* cannabis used in past year	Age [M (SD)]	Gender % female	Joint	Blunt	Pipe	Bong	Total tobacco	Joint	Blunt	Pipe	Bong	Vaporizer	Total non-tobacco	Other^a^
**Europe**																
Austria	1317	750	25.70 (7.49)	23.00	81.0	0.1	0.3	8.0	89.4	3.9	0.1	1.3	1.4	2.0	8.7	2.0
Belgium	2661	1068	25.91 (7.91)	21.80	89.7	0.5	0.0	0.6	90.8	2.9	0.3	1.2	1.3	1.8	7.5	1.9
France	2019	1300	31.19 (11.14)	20.60	83.0	2.0	0.6	1.9	87.5	3.5	1.4	1.3	0.8	4.5	11.5	1.1
Germany	22,232	9905	25.30 (7.84)	19.40	80.2	0.1	0.5	6.4	87.2	4.0	0.3	2.9	2.0	2.2	11.4	1.4
Hungary	3164	1173	27.51 (7.04)	19.40	88.0	0.2	0.6	0.5	89.3	2.6	0.1	4.7	2.3	0.3	10.0	0.7
Republic of Ireland	824	472	26.80 (9.19)	27.20	81.0	0.2	0.0	0.2	81.4	4.2	0.7	6.4	4.2	1.8	17.3	1.3
Denmark	1630	1014	27.36 (9.13)	19.10	81.0	0.4	1.7	3.9	87.0	4.1	0.1	2.9	0.9	3.0	11.0	2.0
Portugal	611	308	25.59 (9.00)	27.20	88.5	1.0	0.0	0.3	89.8	6.8	0.0	1.0	0.3	1.7	9.8	0.3
Spain	1298	820	29.38 (9.83)	24.10	85.4	0.4	0.3	0.3	86.4	7.9	0.5	2.6	0.3	1.1	12.4	1.3
Netherlands	2743	1196	22.38 (5.95)	41.60	86.8	0.2	0.1	0.5	87.6	4.1	0.4	2.0	2.0	1.6	10.1	2.3
Switzerland	4972	1961	27.03 (9.02)	21.30	89.7	0.3	0.1	0.8	90.9	3.0	0.5	1.1	0.8	2.1	7.5	1.6
United Kingdom	7174	3725	27.89 (10.34)	23.80	75.5	0.1	0.1	1.5	77.2	6.0	0.5	6.2	4.4	4.1	21.2	1.7
**Americas**																
Brazil	1065	736	26.39 (8.15)	19.30	6.7	0.3	0.0	0.4	7.4	80.8	2.8	2.1	3.1	2.6	91.4	1.1
United States	6423	4359	32.09 (14.38)	33.10	3.7	0.1	0.3	0.3	4.4	10.7	3.4	48.1	18.7	11.2	92.1	3.5
Canada	834	570	27.83 (11.39)	29.20	10.9	0.4	0.2	4.5	16.0	31.8	0.9	18.7	15.1	13.3	79.8	4.2
Mexico	627	472	26.02 (7.84)	31.30	6.1	0.4	0.4	0.0	6.9	37.8	6.1	40.9	6.7	0.2	91.7	1.3
**Australasia**																
Australia	5789	1947	32.95 (11.87)	28.50	37.0	0.2	2.1	12.3	51.6	15.4	0.3	9.8	12.8	5.8	44.1	4.3
New Zealand	5614	1911	31.48 (11.52)	35.60	17.2	0.1	0.2	3.2	20.7	23.7	0.5	27.9	15.0	3.1	70.2	9.1
Worldwide	70,997	33,687 (47.4%)	27.86 (10.39)	25.86	61.3	0.2	0.5	3.6	65.6	9.5	0.9	11.7	6.0	4.0	32.1	2.4

*^a^Consists of non-tobacco non-inhaled routes of administration (“bucket bong,” “hot knife,” “in food,” “in drink,” and “other”)*.

Globally, tobacco ROAs were more common (65.6%) than non-tobacco ROAs (32.1%). Within the non-tobacco ROA group, 16.3% of the respondents had never tried smoking tobacco independently of cannabis. The most common tobacco ROA was smoking “joints with tobacco” (61.3%); alternative tobacco ROAs were seldom chosen. The most common non-tobacco ROA was “pipe” (11.7%) although “joint” was comparably frequent (9.5%).

Inspection of Table 2 suggests considerable global variation. First, tobacco ROAs were the predominant choice across all European countries (ranging from 90.9% in Switzerland to 77.2% in the UK). Across Europe, frequent adoption of tobacco ROAs was driven by the typical use of “joint with tobacco.” Although a disproportionately greater number of responses were collected from Germany, compared with responses from Portugal, Table 2 indicates a high level of consistency in the tendency to use tobacco ROAs among European countries.

By contrast, in the Americas (Brazil, United States, Canada, and Mexico), the predominant choice is non-tobacco ROAs (88.8% total), ranging from 92.1% in United States to 79.8% in Canada. Within the Americas, there was considerable variation in the most common non-tobacco ROA. “Joint without tobacco” was almost exclusively reported among Brazilian respondents (80.8%), while the other counties tended to use a range of options including “pipe without tobacco” and “bong without tobacco.” Use of vaporizers was only frequent in Canada (13.3%) and the United States (11.2%).

Respondents from Australasia tended to choose a mixture of tobacco and non-tobacco ROAs. Australian respondents were more likely to choose a tobacco ROA (51.6%), mainly consisting of not only “joint with tobacco” (37.0%) but also “bong with tobacco” (12.3%). New Zealand respondents tended to choose a non-tobacco ROA (70.2%) that consisted of a mixture of ROAs, predominantly “pipe without tobacco” (27.9%), “joint without tobacco” (23.7%), and “bong without tobacco” (15.0%).

### Predicting Intention to Use Less Cannabis/Tobacco from ROA

A total of 27.2% of all participants wanted to use less cannabis, 16.1% wanted help to use less cannabis, and 4.6% said they were planning to seek help in the next year (Table 3). For tobacco, 61.1% said that they would like to use less tobacco in the next year, 22.8% stated that they wanted help to use less tobacco in the next 12 months, and 10.2% said they were planning to seek help to use less tobacco in the next 12 months.

**Table 3:** **Binary logistic regressions for ‘‘like to use less’’, ‘‘like help to use less’’, and ‘‘planning to seek help to use less’’, in the next year for cannabis and tobacco**.

	Like to use less		Like help to use less		Planning to seek help to use less	
Variables	aOR	95% CI	aOR	95% CI	aOR	95% CI
**Cannabis**						
Age	0.981^a^	(0.977, 0.985)	1.025^a^	(1.016, 1.034)	1.023^a^	(1.008, 1.039)
Sex	1.108^a^	(1.026, 1.197)	0.870	(0.733, 1.034)	0.970	(0.709, 1.327)
DPM cannabis	1.025^a^	(1.020, 1.030)	1.033^a^	(1.022, 1.045)	1.046^a^	(1.025, 1.068)
DPM tobacco^b^	0.995^a^	(0.992, 0.997)	1.007^a^	(1.000, 1.014)	1.027^a^	(1.013, 1.040)
DPM tobacco with cannabis^b^	1.017^a^	(1.012, 1.023)	1.018^a^	(1.006, 1.030)	0.985	(0.965, 1.006)
ROA	0.626^a^	(0.561, 0.699)	1.615^a^	(1.230, 2.120)	0.849	(0.525, 1.524)
Constant	0.459	–	0.041	–	0.010	–
*N*	18,971		5728		5060	
**Tobacco**						
Age	1.019^a^	(1.015, 1.023)	1.047^a^	(1.041, 1.052)	1.059^a^	(1.052, 1.066)
Sex	1.004	(0.934, 1.080)	0.770^a^	(0.690, 0.858)	0.656^a^	(0.555, 0.775)
DPM cannabis	0.997	(0.992, 1.002)	0.996	(0.988, 1.003)	0.998	(0.986, 1.009)
DPM tobacco^b^	1.034^a^	(1.031, 1.037)	1.045^a^	(1.040, 1.051)	1.049^a^	(1.040, 1.058)
DPM tobacco with cannabis^b^	1.000	(0.995, 1.005)	0.996	(0.989, 1.004)	0.990	(0.979, 1.002)
ROA	1.107^a^	(1.003, 1.221)	1.806^a^	(1.556, 2.095)	2.039^a^	(1.638, 2.539)
Constant	0.519	–	0.033	–	0.009	–
*N*	18,315		11,042		9275	

*DPM, days per month; aOR, adjusted odds ratio; ROA, route of administration (tobacco-based inhaled route is the reference category)*.

*^a^95% CI does not cross 1*.

*^b^Not all respondents had used tobacco or tobacco with cannabis in the last month*.

The odds for “desire to use less cannabis” were 0.625 times lower in the non-tobacco ROA group than in the tobacco ROA group. Conversely, non-tobacco ROAs were associated with a 61.5% increase in odds for “like help to use less cannabis in the next year” in comparison to those using tobacco ROAs. The effects of ROAs on “planning to seek help to use less cannabis” were not significant. Taken together, these results suggest that tobacco ROAs were not consistently associated with levels of motivation to change individuals’ cannabis use.

Among users of both tobacco and cannabis, non-tobacco ROAs were associated with a 10.7% increase in odds for “desire to use less tobacco.” Consistent with this, non-tobacco ROAs were associated with an 80.6% increase in “like help to use less tobacco in the next year” in comparison to tobacco ROAs. Finally, non-tobacco ROAs were associated with a 103.9% increase in the odds for “planning to seek help to use less tobacco.” Together, these results suggest that tobacco ROAs were consistently associated with reduced intention to use less tobacco. This analysis was replicated in those who smoked cannabis >100 days in the past 12 months (see Table S1 in Supplementary Material).

### Exploratory Analysis

#### ROA Associations with Age and Gender

There was a significant association between gender and ROA [χ*^*2*^*(1) = 48.51, *p* < 0.001]. More females used non-tobacco ROAs (36.2%) in comparison to tobacco ROAs (63.8%), and more males used tobacco ROAs (68.2%) in comparison to non-tobacco ROAs (31.8%). Moreover, those using a tobacco ROA (M = 26.23, SD = 8.48) were younger than those using a non-tobacco ROA (M = 30.79, SD = 12.76) [*F*(1, 14,622) = 1058.94, *p* < 0.001].

#### ROA Associations with Drug Use and Impact of Drug Use

Those using a non-tobacco ROA used cannabis on more days per months (M = 13.61, SD = 12.13) in comparison to those using a tobacco-based ROA (M = 12.10, SD = 11.46) [*F*(1, 19,089) = 109.82, *p* < 0.001] and they used more grams per day (M = 0.52, SD = 1.14) than tobacco ROA users (M = 0.42, SD = 0.84) [*F*(112, 556) = 55.05, *p* < 0.001]. Moreover, those using tobacco ROAs (M = 20.76, SD = 11.90) used tobacco more days per month than those using non-tobacco ROAs (M = 13.44, SD = 13.08) [*F*(1, 8501) = 1362.21, *p* < 0.001] and had started using tobacco slightly earlier (M = 14.65, SD = 2.80) than those using non-tobacco ROAs (M = 15.36, SD = 3.26) [*F*(1, 14,149) = 304.62, *p* < 0.001]. There were more negative effects associated with the impact of cannabis in those using a tobacco ROA (M = 3.19, SD = 1.96) in comparison to a non-tobacco ROA (M = 2.52, SD = 1.70) [*F*(1, 19,957) = 846.64, *p* < 0.001]. Participants also found non-tobacco ROAs (M = 7.52, SD = 1.82) more pleasurable than tobacco ROAs (M = 7.11, SD = 1.84) [*F*(1, 20,413) = 356, *p* < 0.001]. Moreover, a comparison between vaporizer users and other non-tobacco ROA users shows that vaporizer users use tobacco on less days per month (M = 9.53, SD = 12.00) than non-tobacco ROA users (M = 13.84, SD = 13.12) [*F*(1, 645) = 58.87, *p* < 0.001].

## Discussion

The aim of this study was to provide a global overview of cannabis and tobacco ROAs and to examine their association with motivation to use less cannabis and tobacco. Our results demonstrate marked global variation in tobacco/non-tobacco ROAs, with distinct patterns across Europe, the Americas, and Australasia. Non-tobacco ROAs were consistently associated with increased motivation to reduce tobacco use, although findings with cannabis were inconsistent. We also found those using tobacco ROAs were more likely to be male and younger than those using non-tobacco ROAs.

Notably, the Americas (Brazil, United States, Canada, and Mexico) had comparatively little use of tobacco ROAs. In North America, there was high use of vaporizers; devices that heat up cannabis electronically, allowing the vapor to be inhaled without combustion (16). The trend toward cannabis vaporizers is significant as they may be less harmful than smoked cannabis (with or without tobacco). They may also be useful for harm reduction for respiratory problems and possibly tobacco use (17–19). Our data suggest a low prevalence of tobacco ROAs and a corresponding higher prevalence of vaporizer use in the United States and Canada, which may be an important predictor of reduced future tobacco consumption among cannabis users in these countries. Indeed, those using vaporizers were using tobacco on fewer days per month in comparison to those using other non-tobacco ROAs in our exploratory analysis.

Recent prevalence statistics show that Oceania, which includes Australasia, has the highest levels of cannabis use (10.3%) (1). Our data suggest in Australia, the process of mixing cannabis and tobacco is used by about half of those smoking cannabis and represents significant nicotine exposure. In New Zealand, on the other hand, tobacco ROAs are less common than non-tobacco ROAs. In comparison to the rest of the world, which tended to have high levels of one route, respondents in Australasian countries use various ROAs. However, we did not receive responses from every country worldwide, and analysis was restricted to countries with 500 or more respondents for reliability considerations. Future studies should aim to recruit from additional countries in order to reflect a “truly global sample.” Moreover, certain forms of coadministration of cannabis and tobacco are strongly governed by cultural norms and ethnicity (particularly in the United States), which might play a role in this association (20, 21) and could be investigated in future research. In this paper, we focused on age and sex, other covariates, such as alcohol, were not our focus, but future research may need to undertake model building approaches to ascertain which demographics should be included.

There are few studies that investigate the effects of ROA, but one recent study found those using “pure” cannabis (equivalent to non-tobacco ROAs in this study) showed less problematic cannabis use than those using cannabis mixed with tobacco (22). Our results are consistent with this and other previous research suggesting tobacco smoking is more problematic for those who also use cannabis (3, 23, 24), and we were also able to adjust for the frequency of cannabis and tobacco use. Our results suggest that tobacco ROAs are associated with a reduced motivation to use less tobacco and more negative effects of cannabis, which may account for the poor tobacco-related cessation reported previously (23, 24). *Post hoc* comparisons between those using non-tobacco ROAs in comparison to those using tobacco ROAs suggest that those using tobacco ROAs are heavier cigarette smokers and started using tobacco earlier. Moreover, only 16% of the present sample were using a non-tobacco ROA and had never tried tobacco suggesting within cannabis users that it is rare to have never tried tobacco.

We also found ROA was not necessarily associated with poor cannabis-related motivations for cessation. An alternative explanation for this finding is that we used a low threshold for cannabis use (once in the last 12 months); however, we did account for the increasing cannabis use in our model which included days per month of cannabis use and predicted motivation to change. Moreover, we replicated the analysis in regular cannabis users (see Table S1 in Supplementary Material). Interestingly, those using a non-tobacco ROA were using cannabis on more days per month, more cannabis per day, and found non-tobacco ROAs more pleasurable, in comparison to those using a tobacco-based ROA, replicating other recent online survey results (25). Practically, this may be related to not having an inexpensive filler to use, but it may also be a factor related to low motivations to use less cannabis. Recent attempts to create cessation programs for co-users seem promising (9, 10); however, in order to tailor tobacco cessation programs for those who smoke cannabis, further emphasis should be on the use of non-tobacco ROAs as this may increase the likelihood and effectiveness of future tobacco quit attempts.

The implications of tobacco ROAs for clinical and public health consequences of cannabis use are significant. Table 4 provides an overview of possible strategies, and their evidence base, for reducing and preventing cannabis and tobacco co-use, as well as directions for future research. The concurrent use of both substances leads to poorer outcomes for cessation attempts than for either drug alone, plays a role in the maintenance of cannabis use, and leads to more significant cannabis withdrawal in isolation (26–28). Concurrent use is associated with synergistic pulmonary harms, and tobacco use significantly increases the risk of malignancy and may independently be associated with an increased risk of developing psychosis (29). Many cultures have adopted non-tobacco ROAs suggesting it is possible to for users to “enjoy” cannabis without tobacco, and it is noteworthy that countries reporting the lowest rates of tobacco ROAs also reported the highest use of vaporizers.

**Table 4:** **Summary of strategies for reducing and preventing cannabis and tobacco co-use and areas and future directions**.

Strategy	Evidence-base
Promote reduction of both simultaneous and co-occurring use	Simultaneous users are 5.1 times more likely to experience cannabis dependence (3, 22) Cigarette smoking alongside cannabis use increases symptoms of cannabis dependence and relapse (27, 41) In comparison to those with cannabis dependence alone, those who are also nicotine dependent have more severe psychosocial and psychiatric outcomes (42)
Promote alternative ROAs, such as vaporizers	Vaporizers may be an acceptable harm reduction intervention to promote as they do less damage to the respiratory system (43). Users rate vaporizers as the most important way of reducing cannabis-related harms (www.globaldrugsurvey.com/brands-highwaycode)
Avoid e-cigarettes	75% of cannabis vaporizer users have also used a nicotine e-cigarette (25). Marketing of cannabis vaporizers alongside e-cigarettes may increase co-use and these devices should be separated at the point of sale
Motivation to change	Administering cannabis without tobacco may increase motivation to reduce tobacco use
Regional variation	Administering cannabis with tobacco is most common in Europe. Dialog between policies to reduce tobacco smoking and those regarding cannabis may be helpful
**Future directions**	
Vaporizers	Further research is required to better define the harm reduction benefits of vaporizers on respiratory health and function as well as potential harms and/or benefits associated with vaporizer use
Harm reduction	Health promotion campaigns should focus on dissociating the use of tobacco and cannabis and should consider differential harm reduction campaigns for cannabis users who smoke cannabis with tobacco
Monitoring	A more accurate description of how cannabis is consumed worldwide through better monitoring and screening tools is required
Controlled experimental studies	Investigating the reasons behind simultaneous use with hypothesis-driven controlled experimental studies including researching the acute psychopharmacological interaction on cognition (8) and reward is warranted

To the best of our knowledge, this is the first global overview of cannabis and tobacco ROAs. Our design afforded us the ability to collect a large sample rapidly and on an unprecedented various ROAs. This methodology has advantages and disadvantages including those surrounding reliability and validity at a population-based level, as discussed elsewhere (30–33). Online surveys are considered a credible vehicle for opportunistic research and are valuable where current data are scarce, as is the current case. These data, therefore, provide a snapshot of the use of cannabis and tobacco ROAs, where there is a paucity of epidemiological data [also see Ref. (16, 25)]. Epidemiological data on the prevalence of certain ROAs, such as vaporization, have yet to be conducted (34), and the GDS has the size and cross-cultural representativeness allowing insight into the changes occurring in cannabis ROAs. Moreover, longitudinal studies are necessary to identify the patterns in co-use over time as cannabis legalization spreads (35, 36).

This study had some limitations. First, we used a self-nominating convenient (drug-using) sample using an Internet survey that this may have some reliability and validity issues that include the limited ability to generalize to the countries included in our analysis (30–33). Therefore, these estimates should be treated with caution until replicated; although our data on UK ROAs show consistency with a previous GDS sample of UK cannabis users (37). Furthermore, the observed consistency within large geographical regions (especially Europe and the Americas) does lend support to genuine global variation; however, our sample was skewed toward people of a young age. Moreover, we did not measure self-reported cannabis dependence and/or tobacco dependence. Cannabis exposure variables can be poor at predicting cannabis use disorders (38), the prevalence of which varies worldwide (39). Furthermore, we focused on our hypotheses regarding cannabis and tobacco co-use and did not consider the role of other poly-drug use, including alcohol, which clearly plays an important role (40) or the role of combinations of ROAs on which there is evidence to suggest the greater the number of ROAs used, the more problematic the cannabis use (22). We modeled three dependent variables each for cannabis and tobacco, which were related to increased motivation to use less of that drug (12); however, these were not clinically validated and can only provide preliminary evidence on motivation to use less of each drug.

## Conclusion

Among a global sample of cannabis users, tobacco ROAs are frequently adopted. This is especially true in European countries, followed by Australasia, and then the Americas, where non-tobacco ROAs are more common. Non-tobacco ROAs were associated with greater motivation to change tobacco use and, therefore, may reduce the harmful consequences of cannabis use.

## Author Contributions

The study was conceived by all authors. The survey was designed by AW. Data were collected by AW. Data were analyzed by CH. The manuscript was prepared by CH. All authors contributed to and have approved the final manuscript.

## Conflict of Interest Statement

AW is the founder of Global Drug Survey. The remaining authors declare that the research was conducted in the absence of any commercial or financial relationships that could be construed as a potential conflict of interest.

## Footnotes

^1^Lawn W, Freeman TP, Pope RA, Joye A, Harvey L, Hindocha C, et al. Acute and chronic effects of cannabinoids on effort-related decision-making and reward learning: an evaluation of the cannabis ‘amotivational’ hypothesis. (Under revision).
