# Supplementary material for: No Smoke without Tobacco: A Global Overview of Cannabis and Tobacco Routes of Administration and Their Association with Intention to Quit
Source: Front Psychiatry. 2016 Jul 5;7:104. doi: 10.3389/fpsyt.2016.00104 (PMC4933835; doi:10.3389/fpsyt.2016.00104)
Supplement: Supplementary file 1 [file Table_1.DOCX]

Supplementary Material

**No Smoke without tobacco - A global overview of cannabis and tobacco routes of administration and their association with intention to quit**

Chandni Hindocha*, Tom P Freeman, Jason A. Ferris, Michael T. Lynskey PhD, Adam R. Winstock PhD.

*** Correspondence:** Corresponding Author: [c.hindocha@ucl.ac.uk](mailto:c.hindocha@ucl.ac.uk)

**Predicting intention to use less cannabis/tobacco among regular users (Supplementary table 1)**

The analysis in section 3.2 was replicated selecting for participants who smoked cannabis >100 days in the last 12 months, in order to investigate ROA associations with motivation to change in regular cannabis users. 14653 participants responded that they had used cannabis in >100 days in the last 12 months (mean age = 28.7; %female = 20.1). 36.3% wanted to use less cannabis, 22.1% wanted help to use less cannabis, and 6.8% said they were planning to seek help in the next year. For tobacco, 64.5% would like to use less tobacco in the next year, 24% wanted help to use less tobacco in the next 12 months and 11.1% were planning to seek help to use less tobacco in the next 12 months.

The odds for ‘desire to use less cannabis’ were 0.377 times *lower* in the non-tobacco ROA group than in the tobacco ROA group for regular cannabis users. Non-tobacco ROAs were associated with a 40.7% increase in odds for ‘like help to use less cannabis in the next year’ in comparison to those using tobacco ROAs. The effects of ROAs on ‘planning to seek help to use less cannabis’ were not significant. This pattern of results replicates the findings in all users.

Non-tobacco ROAs were not significantly associated with ‘desire to use less tobacco’ in those who smoked cannabis >100 days in the last year. Non-tobacco ROAs were associated with a 61.4% increase in ‘like help to use less cannabis in the next year’ in comparison to tobacco ROAs and finally non-tobacco ROAs were associated with a 72.3% increase in the odds for ‘planning to seek help to use less tobacco’.

These results are mostly consistent with the results in section 3.2. In those who use cannabis >100 days per year, non-tobacco ROAs were not associated with desire to use less tobacco, however, this may be a power issue, as the number of respondents was significantly reduced (See Supplementary table 1).

|  | Cannabis | | | | | |
| --- | --- | --- | --- | --- | --- | --- |
|  | **Like to use less** | | **Like *help* to use less** | | **Planning to seek help to use less** | |
| **Variables** | aOR | 95% CI | aOR | 95% CI | aOR | 95% CI |
| Age | 0.981^a^ | [0.976, 0.986] | 1.026^a^ | [1.015, 1.037] | 1.024^a^ | [1.006, 1.042] |
| Sex | 0.963 | [0.862, 1.076] | 0.84 | [0.689, 1.024] | 0.91 | [0.639, 1.296] |
| DPM cannabis | 1.003 | [0.997, 1.010] | 1.017^a^ | [1.004, 1.030] | 1.028^a^ | [1.005, 1.051] |
| DPM tobacco^b^ | 0.989^a^ | [0.985, 0.993] | 1.005 | [0.998, 1.013] | 1.027^a^ | [1.011, 1.043] |
| DPM tobacco with cannabis^b^ | 1.002 | [0.996, 1.009] | 1.011 | [0.998, 1.024] | 0.971 | [0.951, 1.993] |
| ROA | 0.377^a^ | [0.321, 0.444] | 1.407^a^ | [1.001, 1.978] | 0.652 | [0.346, 1.230] |
| Constant | 1.560 | - | 0.081 | - | 0.025 |  |
| N | 14653 |  | 3546 |  | 2979 |  |
|  | Tobacco | | | | | |
|  | **Like to use less** | | **Like *help* to use less** | | **Planning to seek help to use less** | |
| **Variables** | aOR | 95% CI | aOR | 95% CI | aOR | 95% CI |
| Age | 1.019^a^ | [1.013, 1.024] | 1.045^a^ | [1.038, 1.052] | 1.056^a^ | [1.046, 1.065] |
| Sex | 0.95 | [0.847, 1.066] | 0.816^a^ | [0.694, 0.958] | 0.623^a^ | [0.490, 0.793] |
| DPM cannabis | 0.986^a^ | [0.980, 0.993] | 0.994 | [0.985, 1.004] | 0.993 | [0.979, 1.008] |
| DPM tobacco^b^ | 1.024^a^ | [1.020, 1.029] | 1.039^a^ | [1.030, 1.047] | 1.047^a^ | [1.033, 1.061] |
| DPM tobacco with cannabis^b^ | 1.002 | [0.996, 1.099] | 0.992 | [0.983, 1.002] | 0.99 | [0.976, 1.004] |
| ROA | 1.149 | [0.983, 1.342] | 1.614^a^ | [1.304, 1.996] | 1.723^a^ | [1.251, 2.372] |
| Constant | 0.869 | - | 0.046 | - | 0.012 | - |
| N | 8612 |  | 5441 |  | 4487 |  |
| Notes: DPM -days per month, aOR- adjusted odds ratio, ROA - route of administration (tobacco-based inhaled route is the reference category), ^a^ - 95% CI does not cross 1. ^b^- Not all respondents had used tobacco or tobacco with cannabis in the last month. | | | | | | |

**Supplementary table 1:** Binary logistic regressions for like to use less, like help to use less and planning to seek help to use less, in the next year for cannabis and tobacco for people who smoke cannabis > 100 days in the last 12 months.
